# Occupational Therapy in Return‐to‐Work Processes After Stroke: A Scoping Review of Ongoing Therapeutic Support and Implications for Work–Treatment Balance

**DOI:** 10.1155/oti/4254501

**Published:** 2026-07-24

**Authors:** Reiko Miyamoto, Peter Bontje

**Affiliations:** ^1^ Division of Occupational Therapy, Faculty of Health Sciences, Tokyo Metropolitan University, Tokyo, Japan, tmu.ac.jp; ^2^ Department of Rehabilitation, Faculty of Health Sciences, Hiroshima Cosmopolitan University, Hiroshima, Japan

**Keywords:** occupational therapy, return to work, scoping review, stroke, work–treatment balance

## Abstract

**Background:**

Return to work (RTW) after stroke is a complex process influenced by residual impairments, workplace demands, and rehabilitation support. However, how therapeutic support is structurally incorporated into RTW processes and the specific contributions of occupational therapy (OT) remain unclear.

**Objective:**

This scoping review is aimed at examining how therapeutic support is incorporated into RTW processes following stroke and at clarifying the roles of OT within these support structures.

**Methods:**

Comprehensive literature searches were conducted in Ichushi‐Web, PubMed, and Web of Science, complemented by manual searches. Studies published in Japanese or English up to February 2026 were included if they addressed stroke survivors and work participation–related support. Data were extracted using a structured framework to map therapeutic integration during RTW and OT roles. Facilitators, barriers, and assessment approaches were also summarized.

**Results:**

Fifty‐eight studies met the inclusion criteria. Therapeutic support was described at different stages of the RTW process, ranging from interventions completed before work resumption to support provided alongside RTW and support integrated with workplace demands. OT roles were identified across 10 categories, most commonly involving work participation–oriented assessment and job analysis, employer liaison and stakeholder meetings and mediation, and workplace evaluation and accommodations. Functional impairments and fatigue were frequently reported as barriers to sustained work participation; however, fatigue‐specific assessments were reported less frequently.

**Conclusions:**

Therapeutic integration within ongoing work participation is a key structural feature of RTW support for stroke survivors and may also be relevant to how work–treatment balance (WTB) is addressed in practice. The findings highlight the diverse roles of OT in linking worker capacity, workplace demands, and service coordination. They also suggest a discrepancy between commonly reported barriers, particularly fatigue, and the assessment approaches used in RTW‐focused studies.

## 1. Introduction

Return to work (RTW) is increasingly understood as a dynamic process that unfolds alongside ongoing health management, rather than simply a rehabilitation outcome [1]. Policy‐oriented evidence has also highlighted the broader health benefits of work participation [2]. For individuals with acquired brain injuries, including stroke, functional impairments have been identified as prognostic factors for RTW [3]. This suggests that therapeutic support may need to extend into, rather than conclude before, the RTW process. Research on vocational rehabilitation has highlighted a gap between medical rehabilitation and actual workplace participation, emphasizing the need for coordinated and sustained RTW interventions [4, 5].

Systematic reviews have examined RTW after stroke, focusing on prognostic factors and employment outcomes [6, 7]. Other reviews have evaluated vocational rehabilitation and multidisciplinary interventions, providing valuable evidence regarding RTW outcomes and intervention effectiveness [8]. However, these studies often conceptualize RTW as an endpoint or discrete intervention target. The structural integration of ongoing therapeutic support within RTW trajectories remains insufficiently examined. Specifically, there is a need to understand how ongoing therapeutic support is organized in relation to work participation—whether treatment precedes, parallels, or is integrated with RTW processes. In this review, therapeutic support was used as a broad term encompassing rehabilitation, assessment, education, workplace‐focused interventions, and other therapeutic activities reported in relation to RTW after stroke. Occupational therapy (OT), which focuses on the interaction between person, environment, and occupation [9, 10], is particularly relevant in contexts requiring coordination between therapeutic management and workplace participation. However, how OT roles are enacted across different levels of therapeutic integration during RTW has not been comprehensively examined.

In Japan, the Ministry of Health, Labour and Welfare introduced the concept of work–treatment balance (WTB) to describe support enabling individuals to maintain employment while receiving appropriate medical care [11]. Although policy‐specific, the challenge of integrating treatment with sustained work participation resonates internationally. In this review, WTB serves as an analytical lens to interpret patterns of therapeutic integration within RTW processes and to clarify OT′s potential contribution to sustained work participation. WTB was therefore used as an interpretive framework rather than a formal outcome or classification variable. Because WTB is not consistently operationalized in the international literature, this review indirectly examines WTB by exploring how therapeutic support is structurally integrated within the RTW pathway. This scoping review (ScR) is therefore aimed at mapping how therapeutic support is incorporated into RTW support processes after stroke and at identifying OT‐related roles reported within these RTW support processes.

## 2. Materials and Methods

### 2.1. Research Design

This study employed a ScR methodology to map and categorize how therapeutic support is incorporated into RTW processes following stroke, with a focus on ongoing therapeutic involvement and OT‐related roles. A ScR was chosen to explore the range and structural patterns of existing evidence, clarify the conceptual positioning of therapeutic support within RTW processes, and identify knowledge gaps, rather than to evaluate intervention effectiveness [12, 13]. The review was conducted in accordance with the Preferred Reporting Items for Systematic Reviews and Meta‐Analyses extension for ScRs [14].

To guide eligibility criteria and data extraction, the Population–Concept–Context framework was applied [15]. The population included working‐age adults who had experienced a stroke and were either preparing for or actively engaged in RTW processes. The concept focused on therapeutic support provided in relation to RTW processes, with particular attention to both the level of therapeutic integration and the OT‐related roles within these processes. The context encompassed RTW support delivered in healthcare and/or workplace settings, reported in either Japanese or English, regardless of the country of study. Based on this Population–Concept–Context framework, the following eligibility criteria were operationalized.

### 2.2. Research Questions (RQs)

The following RQs were derived from the study objectives:

RQ1. How is therapeutic support incorporated into RTW support processes after stroke?
RQ2. What OT‐related roles are reported within these RTW support processes?
RQ3. What facilitators and barriers to RTW are reported in the literature?

### 2.3. Identification of Relevant Literature

#### 2.3.1. Search Strategy

To identify studies pertaining to therapeutic support within RTW processes following stroke, a comprehensive search strategy was developed [15]. Electronic database searches were conducted in Ichushi‐Web (Japanese database), PubMed, and Web of Science Core Collection on February 7, 2026. There were no restrictions placed on publication year, publication type, or open‐access status; studies published in either Japanese or English were eligible for inclusion.

The search strategy was structured around two primary concept blocks: (1) stroke‐related terms and (2) RTW‐ and work participation–related terms. Within each concept, synonyms were combined using “OR,” and the two blocks were combined using “AND.” Given that therapeutic integration within RTW processes is not consistently indexed as a distinct concept, a broad RTW‐related search strategy was adopted, and eligibility was determined during screening. For Ichushi‐Web, both thesaurus terms (/TH) and free‐text terms were used. Searches were conducted in Japanese, using terms such as “脳卒中” (stroke), “脳血管障害” (cerebrovascular disease), “両立支援” (WTB support), and “復職” (RTW). For PubMed, Medical Subject Headings (MeSH) and free‐text terms were applied. Stroke‐related terms included “stroke” (MeSH), stroke, “cerebrovascular accident,” and CVA. Work‐related terms, such as “return‐to‐work,” “vocational rehabilitation,” “work reintegration,” and “employment support,” were primarily searched within the title and abstract fields.

In addition to electronic database searches, manual searches were performed on selected Japanese journals. To correspond with the period during which structured WTB initiatives began to emerge in Japan [16], the manual search was limited to articles published from 2010 onward. No year restriction was applied to the electronic database searches to ensure comprehensive international coverage. Furthermore, the reference lists of included studies and relevant reviews were screened to identify any additional eligible articles. Detailed search strategies for each database are presented in Table 1.

**Table 1:** Search strategy for each database.

Database	Date searched	Search strategy summary	Limits applied
PubMed	Feb. 7, 2026	((“Stroke”[MeSH] OR stroke[tiab] OR “cerebrovascular accident”[tiab] OR CVA[tiab]) AND (“return to work”[tiab] OR “vocational rehabilitation”[tiab] OR “work reintegration”[tiab] OR “employment support”[tiab])) AND (“0001/01/01”[Date ‐ Publication]: “2026/02/07”[Date ‐ Publication])	None
Web of Science Core Collection	Feb. 7, 2026	TS = (stroke OR poststroke OR “post‐stroke” OR “cerebrovascular accident” OR CVA OR “brain infarction” OR “cerebral infarction” OR “intracerebral hemorrhage”) AND TS = (“return to work” OR “return‐to‐work” OR RTW OR employment OR “return to employment” OR “work participation” OR “work reintegration” OR “vocational rehabilitation” OR “occupational rehabilitation” OR “employment support” OR “work ability” OR “work capacity” OR “work retention” OR “work resumption”)	None
Ichushi‐Web	Feb. 7, 2026	(“治療と仕事の両立支援” OR “両立支援” OR “復職支援” OR 復職 OR “職場復帰” OR “就労支援”) AND (“脳卒中” OR “脳血管障害”) ∗画面設定:フィールド・TH・絞り込みなし	None
Hand search	Feb. 7, 2026	・*Journal of Occupational Health* ・*Japanese Journal of Vocational Rehabilitation*	2010–now

#### 2.3.2. Eligibility Criteria

Studies were eligible if they focused on stroke survivors, either exclusively or within mixed populations where stroke‐specific data could be distinguished, and addressed work participation or RTW‐related support, including vocational rehabilitation or WTB‐related coordination. Eligible studies examined support structures, processes, interventions, professional roles, or stakeholder perspectives related to work participation and reported original empirical findings or detailed descriptions of support implementation, including quantitative, qualitative, and mixed‐methods studies, case reports, or practice‐based reports. Studies were excluded if they did not report extractable stroke‐specific findings, focused only on medical recovery or symptom assessment without addressing work participation, or were review articles, editorials, commentaries, or other publications lacking primary data or detailed descriptions of support processes.

### 2.4. Study Selection

**Figure 1:**
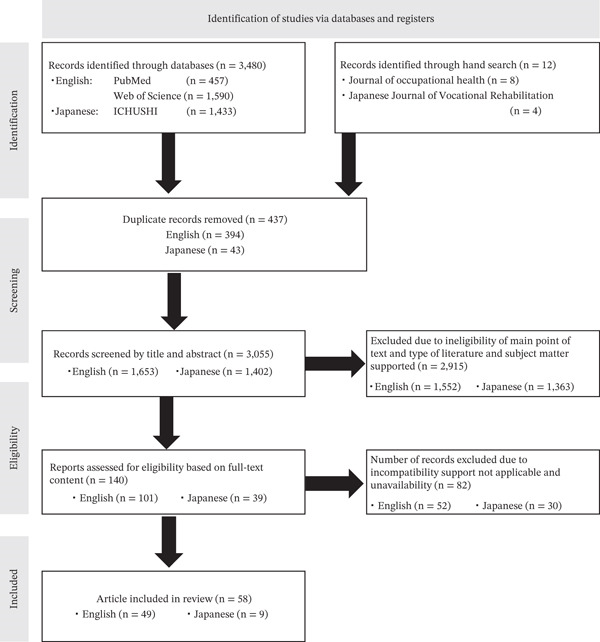
Flow of study identification through databases and registers.

All retrieved records were exported from each database and imported into Rayyan (Qatar Computing Research Institute), a web‐based systematic review management platform. Duplicate entries were first removed automatically using Rayyan and then verified manually. Title and abstract screening was conducted independently by the first and second authors, and full texts of potentially eligible articles were retrieved for independent assessment by the same reviewers. Any disagreements during the screening process were resolved through discussion until consensus was reached. The study selection process is illustrated in the Preferred Reporting Items for Systematic Reviews and Meta‐Analysis flow diagram (Figure 1).

### 2.5. Data Extraction

Data were extracted using a structured extraction form developed for this review. The extraction framework was piloted and refined during an initial phase of charting. Extracted information included study identification number; research type and country, research objectives, perspective (such as survivor, employer, or healthcare professional), members involved in providing support, methods, assessment tools, OT roles, multidisciplinary activities potentially delivered by occupational therapists (OTs), Person–environment–occupation (PEO) classification, ongoing therapeutic interventions during RTW, results (category and details), and factors facilitating or hindering RTW. OT roles were extracted only when explicitly attributed to OTs in the original studies. Activities described as part of general RTW coordination or multidisciplinary rehabilitation were not classified as OT roles unless OT involvement was clearly specified. When professional attribution was unclear, such as when activities were described under a general RTW coordinator role without specifying OT involvement, the OT role was coded as “none.” This approach was adopted to prevent overattribution of OT roles. Extracted data was summarized in structured tables, with the full data extraction table provided in Supporting Information.

### 2.6. Data Analysis and Synthesis

Data analysis followed the predefined RQs and employed a descriptive and iterative mapping approach, consistent with the ScR methodology. Extracted data was first summarized to provide an overview of general study characteristics, including country, research design, perspective, and key features of RTW‐related support. Study designs were documented to describe the methodological characteristics of the included literature; however, formal quality appraisal of individual studies was not undertaken, consistent with ScR objectives.

#### 2.6.1. Classification of Support Contents

Support activities were categorized into three domains based on the PEO model [9, 17], which conceptualizes occupational performance as the dynamic and transactional interaction among personal factors (e.g., skills, abilities, and health status), environmental contexts (e.g., physical, social, and institutional conditions), and occupational demands. This classification aligns with the World Health Organization′s International Classification of Functioning, Disability and Health framework [18], which views work participation as the result of interactions among body functions, activities, participation, and environmental factors.

Representative activities related to personal factors included functional capacity and performance assessments, cognitive demands, and job matching. Environmental factors encompassed workplace modification or accommodations, employer liaison, and occupational health collaboration. Occupational factors involved work task analysis, self‐management training, role reconstruction and life redesign, and graded RTW planning.

#### 2.6.2. Classification of Therapeutic Intervention During RTW

To examine how therapeutic support was positioned in relation to work participation, studies were classified into four levels based solely on explicit textual descriptions: therapeutic support during RTW not describing treatment completed before RTW, treatment continued in parallel with RTW, and treatment explicitly integrated with workplace demands. The latter two levels—parallel support and integrated support—were considered structural forms of WTB, as they involved ongoing therapeutic support during active work participation. “Parallel support” referred to situations in which therapeutic interventions continued alongside the RTW process but were not explicitly linked to job demands, workplace activities, or employer coordination. In contrast, integrated support described interventions in which therapeutic activities were clearly connected to work‐related tasks, workplace demands, employer involvement, or structured RTW programs designed to facilitate work participation.

Classification decisions relied entirely on what was explicitly reported in each study, without interfering with integration where it was not clearly reported. For example, studies describing early or acute‐phase RTW support were categorized as “therapeutic support during RTW not described” if the structure of therapeutic intervention during the RTW process was unclear, even when policy‐level or conceptual descriptions suggested integrated support.

#### 2.6.3. Classification of RTW Facilitators, Barriers, and Assessment Measures

Facilitators, barriers, and assessment measures reported in the included studies were inductively categorized according to content, with similar items combined into broader thematic categories. Mapping reported challenges alongside the types of assessment approaches allowed identification of potential gaps in the evaluation and support of stroke survivors returning to work.

#### 2.6.4. Cross‐Tabulation

For RQ2, OT‐related roles were synthesized descriptively using only studies where OT involvement was explicitly reported. Recurring OT‐related activities were identified and inductively grouped into thematic role categories and cross‐tabulated with PEO domains and therapeutic integration levels to examine patterns of OT role distribution.

#### 2.6.5. Researcher Reflexivity

The analysis was conducted by two researchers with over 10 years of clinical experience as OTs and more than 20 years of experience in education. Both are involved in teaching and research on RTW support and acknowledged that their professional perspectives could influence data categorization and interpretation. To enhance transparency and analytic rigor, the extraction framework and classification criteria were clearly defined, and all analytic decisions were continuously refined through discussions between the researchers throughout the analysis process.

Generative artificial intelligence (AI) tools (ChatGPT and OpenAI) were used solely for language editing and refinement of the manuscript. These tools were not involved in the study design, data collection, data analysis, or interpretation of the results.

## 3. Results

### 3.1. Descriptive Mapping of Included Studies

The search process identified a total of 3492 records, of which 3480 were retrieved from electronic database searches (2047 in English and 1433 in Japanese) and 12 additional records were obtained through manual searches of a relevant Japanese journal. After removing duplicates and screening for eligibility, 58 articles were included in the final analysis (Figure 1 and Table 2).

**Table 2:** Included studies and their key details for analysis.

No.	Country	First author	Year	Title	Journal	Volume	Issue	Page
1	USA	Kempers, E.	1994	Preparing the Young Stroke Survivor for Return to Work	*Topics in Stroke Rehabilitation*	1	1	65–73
2	USA	Koch, L	2005	Returning to Work After the Onset of Illness: Experiences of Right Hemisphere Stroke Survivors	*Rehabilitation Counseling Bulletin*	48	4	209–218
3	UK	Lock, S	2005	Work After Stroke: Focusing on Barriers and Enablers	*Disability & Society*	20	1	33–47
4	SE	Medin, J	2006	Stroke Patients′ Experiences of Return to Work	*Disability and Rehabilitation*	28	17	1051–1060
5	JP	Katsuo Taya	2007	Practical Stroke Rehabilitation: Specific Approaches to Facilitating Return to Work in the Late Recovery and Chronic Phases From a Vocational Rehabilitation Perspective	*Medical Rehabilitation*	0	85	231–236
6	UK	Alaszewski, A	2007	Working After a Stroke: Survivors′ Experiences and Perceptions of Barriers to and Facilitators of the Return to Paid Employment	*Disability and Rehabilitation*	29	24	1858–1869
7	UK	Gilworth, G	2009	Personal Experiences of Returning to Work Following Stroke: An Exploratory Study	*WORK: A Journal of Prevention, Assessment & Rehabilitation*	34	1	95–103
8	USA	Culler, KH	2011	Barriers and Facilitators of Return to Work for Individuals With Strokes: Perspectives of the Stroke Survivor, Vocational Specialist, and Employer	*Topics in Stroke Rehabilitation*	18	4	325–340
9	USA	Hartke, RJ	2011	Critical Factors Related to Return to Work After Stroke: A Qualitative Study	*Topics in Stroke Rehabilitation*	18	4	341–351
10	ZA	Ntsiea, MV	2012	Return to Work Services Rendered for Patients at Stroke Rehabilitation Facilities in Gauteng Province, South Africa	*International Journal of Therapy and Rehabilitation*	19	3	130–134
11	JP	Saito, Y	2013	Work Support for Working Age Persons Who Have Experienced a Stroke in Japan: Cooperation Between Hospitals and Work Support Agencies	*WORK: A Journal of Prevention, Assessment & Rehabilitation*	45	2	267–272
12	SE	Vestling, M	2013	Thoughts and Experiences From Returning to Work After Stroke	*WORK: A Journal of Prevention, Assessment & Rehabilitation*	45	2	201–211
13	UK	Coole, C	2013	Returning to Work After Stroke: Perspectives of Employer Stakeholders, a Qualitative Study	*Journal of Occupational Rehabilitation*	23	3	406–418
14	UK	Sinclair, E	2014	Developing Stroke‐Specific Vocational Rehabilitation: A Soft Systems Analysis of Current Service Provision	*Disability and Rehabilitation*	36	5	409–417
15	UK	Grant, M	2014	Return to Work After Stroke: Recording, Measuring, and Describing Occupational Therapy Intervention	*British Journal of Occupational Therapy*	77	9	457–465
16	JP	Kondo, D	2015	The Current Situation and Problems of Re‐Employment Support in the Acute Phase	*Japanese Society of Occupational Medicine and Traumatology*	63	6	343–350
17	ZA	Ntsiea, MV	2015	The Effect of a Workplace Intervention Programme on Return to Work After Stroke: A Randomised Controlled Trial	*Clinical Rehabilitation*	29	7	663–673
18	USA	Hartke, RJ	2015	Survey of Survivors′ Perspective on Return to Work After Stroke	*Topics in Stroke Rehabilitation*	22	5	326–334
19	JP	Toyota, A	2016	Content and Current Status of a Workplace‐Life Balance Support Project in the Stroke Rehabilitation Field: 2015 Progress	*Japanese Society of Occupational Medicine and Traumatology*	64	4	208–212
20	SE	Hellman, T	2016	Return to Work After Stroke: Important Aspects Shared and Contrasted by Five Stakeholder Groups	*WORK: A Journal of Prevention, Assessment & Rehabilitation*	55	4	901–911
21	UK	Balasooriya‐Smeekens, C	2016	Barriers and Facilitators to Staying in Work After Stroke: Insight From an Online Forum	*BMJ Open*	6	4	
22	JP	Toyota, A	2017	Dual Support Model of Treatment and Working Life for Stroke Patients: Roles and Functions of Return to Work Coordinators From Hospital to Work	*Japanese Journal of Vocational Rehabilitation*	30	2	12–20
23	SE	Nilsson, AÖ	2017	Experiences of the Return to Work Process After Stroke While Participating in a Person‐Centred Rehabilitation Programme	*Scandinavian Journal of Occupational Therapy*	24	5	349–356
24	SE	Norstedt, M	2017	The (Im)possibilities of Returning to Work After a Stroke	*WORK: A Journal of Prevention, Assessment & Rehabilitation*	56	4	637–647
25	NG	Soeker, MS	2017	Exploring the Experiences of Rehabilitated Stroke Survivors and Stakeholders With Regard to Returning to Work in South‐West Nigeria	*WORK: A Journal of Prevention, Assessment & Rehabilitation*	57	4	595–609
26	USA	Scott, SL	2018	Return to Work After Stroke: A Survey of Occupational Therapy Practice Patterns	*Occupational Therapy in Health Care*	32	3	195–215
27	SE	Palstam, A	2018	Experiences of Returning to Work and Maintaining Work 7 to 8 Years After a Stroke: A Qualitative Interview Study in Sweden	*BMJ Open*	8	7	
28	JP	Kato, G	2019	A Role of Acute Hospital for Treatment and Work‐Life Balance Support in Stroke Patients ‐ Consideration From Results of Satisfaction Survey	*Japanese Society of Occupational Medicine and Traumatology*	67	3	175–180
29	SE	Gard, G	2019	Need for Structured Healthcare Organization and Support for Return to Work After Stroke in Sweden	*Journal of Rehabilitation Medicine*	51	10	741–748
30	JP	Fukushima, T	2020	Occupational Therapy Under the Promotion of Health and Employment Support for Stroke Patients ~A Case of Left Hemiplegia and Attention Disorder Following a Subarachnoid Hemorrhage Complicated by Cerebral Infarction	*Japanese Society of Occupational Medicine and Traumatology*	68	3	194–198
31	JP	Kato, G	2020	Current Status and Problems of Health and Employment Support for Stroke Patients Discharged From the Acute Hospital to Their Own Homes Without Transferring to Other Hospitals	*Japanese Society of Occupational Medicine and Traumatology*	68	6	361–365
32	UK	Balasooriya‐Smeekens, C	2020	How Primary Care Can Help Survivors of Transient Ischaemic Attack and Stroke Return to Work: Focus Groups With Stakeholders From a UK Community	*British Journal of General Practice*	70	693	E294–E302
33	SE	Nilsson, AO	2020	Being a Co‐Worker or a Manager of a Colleague Returning to Work After Stroke: A Challenge Facilitated by Cooperation and Flexibility	*Scandinavian Journal of Occupational Therapy*	27	3	213–222
34	SG	Bin Zainal, MN	2020	Supporting People With Stroke to Return to Work in Singapore: Findings From a Pilot Vocational Rehabilitation Program	*American Journal of Occupational Therapy*	74	6	
35	JP	Onose, T	2021	Return‐to‐Work Support in Occupational Therapy for Recovery	*The Japanese Journal of Occupational Therapy*	55	10	1136–1140
36	ZA	Dreyer, G	2021	Vocational Rehabilitation for Young Stroke Survivors in Gauteng Public Healthcare: Occupational Therapists′ Perceptions	*WORK: A Journal of Prevention, Assessment & Rehabilitation*	69	1	91–107
37	SE	Johansson, U	2021	The Delivery of the ReWork‐Stroke Program: A Process Evaluation	*WORK: A Journal of Prevention, Assessment & Rehabilitation*	70	2	467–478
38	SG	O′Keefe, S	2021	Designing an Intervention Process That Embeds Work‐Focussed Interventions Within Inpatient Rehabilitation: An Intervention Mapping Approach	*Australian Occupational Therapy Journal*	68	1	65–77
39	SE	Johansson, U	2021	The ReWork‐Stroke Rehabilitation Programme Described by Use of the TIDieR Checklist	*Scandinavian Journal of Occupational Therapy*	28	5	375–383
40	JP	Toyota, A	2022	Examining Return‐to‐Work Status of Stroke Patients Using a Database of the Health and Employment Support Coordinator	*Japanese Journal of Stroke*	44	3	259–267
41	AU	Turner, A	2022	Feasibility of the Community‐Based Stay at Work Intervention (SAWI) for Stroke Survivors	*Journal of Vocational Rehabilitation*	57	2	151–164
42	SE	Lindgren, I	2022	Work Conditions, Support, and Changing Personal Priorities Are Perceived Important for Return to Work and for Stay at Work After Stroke ‐ A Qualitative Study	*Disability and Rehabilitation*	44	11	2500–2506
43	NZ	Martin, RA	2023	Early Opportunities to Explore Occupational Identity Change: Qualitative Study of Return‐to‐Work Experiences After Stroke	*Journal of Rehabilitation Medicine*	DOI: 10.2340/jrm.v55.4825		1–13
44	SG	Mohamad, NBZ	2023	A Qualitative Study of Singaporean Perspectives on Returning to Work After Stroke	*WORK: A Journal of Prevention, Assessment & Rehabilitation*	75	2	541–552
45	ZA	Soeker, MS	2024	The Usefulness of the Model of Occupational Self Efficacy (MOOSE) in Returning Stroke Survivors to Work in a Rural Community in Cape Town, South Africa	*Journal of Vocational Rehabilitation*	60	3	341–352
46	SG	Mohamad, NB	2024	Experiences of Participating in a Vocational Rehabilitation Program in Singapore	*Disability and Rehabilitation*	46	1	139–149
47	AU	Moore, N	2024	“I′ve still got a job to go back to”: The Importance of Early Vocational Rehabilitation After Stroke	*Disability and Rehabilitation*	46	13	2769–2776
48	UK	Clarke, DJ	2024	The RETurn to work After stroKE (RETAKE) Trial: Findings From a Mixed‐Methods Process Evaluation of the Early Stroke Specialist Vocational Rehabilitation (ESSVR) Intervention	*PLOS One*	19	10	
49	AU	Lanyon, L	2024	Until You Are in the Chair and Executing Your Role, You Do Not Know’: A Qualitative Study of the Needs and Perspectives of People With Stroke‐Related Communication Disabilities When Returning to Vocational Activity	International Journal of Language & Communication Disorders	59	6	2655–2670
50	CN	Liu, ZW	2025	Understanding the Needs of Young and Middle‐Aged Chinese People Who Have Experienced a Stroke Who Have Not Successfully Returned to Work: A Qualitative Study	*Health Expectations*	28	1	
51	UK	Craven, K	2025	What Do Employers Need When Supporting Stroke Survivors to Return to Work?: A Mixed‐Methods Study	*Topics in Stroke Rehabilitation*	32	4	392–404
52	SG	Chen, NYC	2025	Perceived Benefits and Challenges of a Work Skills Training Workshop After Stroke: A Qualitative Study With Stroke Survivors and Caregivers	*Disability and Rehabilitation*	47	11	2820–2827
53	SG	Wei, EFJ	2025	Evaluation of the SLS CHARTER Care Model to Support Post‐Stroke Care Continuity and Employment	*Scientific Reports*	15	1	
54	USA	Riley, E	2025	Barriers and Facilitators Impacting Return‐to‐Work Reported by People With Poststroke Aphasia	*Seminars in Speech and Language*	0	0	
55	HK	Lo, SHS	2025	Young Stroke Survivors′ Experiences and Approaches to Community Reintegration: A Descriptive Qualitative Study	*BMC Public Health*	26	1	
56	SE	Vollertsen, J	2025	Return to Work With Fatigue After Stroke: A Complex Occupational Adaptation Process	*Scandinavian Journal of Occupational Therapy*	32	1	
57	UK	Trusson, D	2025	Experiences of Support to Return to Work After Stroke: Longitudinal Case Studies From RETAKE Trial	*Health Technology Assessment*	DOI: 10.3310/WRKS9661		1–27
58	SG	Chen, N	2026	Stakeholder Perspectives on a Return‐to‐Work Cognitive Intervention After Stroke	*Clinical Rehabilitation*	DOI: 10.1177/02692155251414362		1–13

The included studies employed a variety of methodological designs, including qualitative studies (*n* = 34), mixed‐methods studies (*n* = 5), quantitative observational studies (*n* = 7), quantitative experimental studies (*n* = 1), process and implementation evaluation studies (*n* = 3), feasibility or pilot studies (*n* = 3), and conceptual, development, or descriptive reports (*n* = 5).

Publication patterns differed between Japanese and international studies. Japanese studies were published from 2007 onward, whereas international studies dated back to 1994, although only one study was published in each of those early years. More consistent publication trends emerged around 2013 in Japan and around 2005 internationally, indicating a gradual increase in research on RTW support after stroke over time (Table 2).

A temporal trend was also observed in the development of RTW support programs. Following the publication of the Early Stroke Specialist Vocational Rehabilitation program in 2014, several studies reported initiatives targeting earlier stages of recovery, including programs implemented within primary care or early rehabilitation contexts.

Several studies have described specific RTW support programs. Across the included literature, 12 distinct RTW programs were identified. Among these, the Early Stroke Specialist Vocational Rehabilitation program (*n* = 3) and the Model of Occupational Self‐Efficacy (*n* = 2) were reported in multiple studies.

### 3.2. Therapeutic Integration Patterns in RTW Support (RQ1)

Therapeutic integration was variably described across the included studies. Among the studies that reported sufficient information, therapeutic support most commonly continued in parallel with RTW or was explicitly integrated with workplace demands. In contrast, a smaller number of studies described therapeutic interventions that were completed before RTW, while some studies did not provide sufficient information regarding the timing or continuity of therapeutic involvement. Therapeutic support continued in parallel with RTW in 10 studies and was explicitly integrated with workplace demands in 12 studies. In contrast, treatment was reported to be completed before RTW in 13 studies, whereas 23 studies did not provide sufficient information regarding therapeutic continuity.

### 3.3. OT Roles Within RTW Support Processes (RQ2)

Among the 58 included studies, 35 (60.3%) reported OT‐related activities and were therefore included in the analysis of OT roles (Table S1). Three studies explicitly identified OTs as the primary professionals responsible for RTW support (Nos. 15, 36, and 45), and three studies described OTs in coordinator roles, such as RTW coordinators or WTB coordinators (Nos. 23, 37, and 39). OT‐related activities were grouped inductively into 10 role categories based on similarities in intervention focus and target domains. To improve interpretability, these categories were further organized into five broader domains reflecting functional areas of OT involvement in RTW support: work capacity assessment, work‐focused therapeutic intervention, workplace interface and environmental adaptation, RTW process coordination, and psychosocial support. Because individual studies often reported multiple OT activities, a single study could contribute to more than one role category. The distribution of these roles across PEO domains and levels of ongoing therapeutic integration is summarized in Table 3.

**Table 3:** Reported occupational therapy roles across domains and levels of therapeutic integration (*n* = 40 studies).

Domain	No.	Role category	PEO classification			Ongoing therapeutic intervention during RTW				*N*
			P	E	O	Not described	Completed	Continued	Integrated	
Work capacity assessment	1	Work participation–oriented assessment and job analysis	20	22	24	6	6	4	8	24
Work‐focused therapeutic intervention	2	Work simulation, work training, and work conditioning	13	17	17	2	6	4	5	17
	3	Cognitive‐communication strategies and compensatory skills training	7	8	8	0	1	3	4	8
	4	Fatigue/energy management and work self‐management	4	6	6	0	1	2	3	6
Workplace interface and environmental adaptation	5	Workplace evaluation and accommodations	16	19	19	4	3	4	8	19
	6	Employer liaison, stakeholder meetings, and mediation	17	20	20	5	2	5	8	20
RTW process coordination	7	RTW planning, graded return, and follow‐up monitoring	11	14	16	2	4	3	7	16
	8	Case coordination and cross‐sector service navigation	10	13	13	3	1	4	5	13
	9	Education and resource/benefits navigation	7	10	12	1	4	2	5	12
Psychosocial support	10	Psychosocial and identity/self‐efficacy support	7	9	10	2	3	2	3	10
		Total	105	129	135	23	28	31	53	

*Note:* Studies contribute to multiple role categories; column totals do not equal the number of studies (*n*) in each model, as one study falls into multiple.

Abbreviations: E = environment, O = occupation, P = person, RTW = return to work.

The most frequently reported OT activity was “work participation–oriented assessment and job analysis” (Category 1, *n* = 24), followed by “employer liaison, stakeholder meetings, and mediation” (Category 6, *n* = 20), “workplace evaluation and accommodations” (Category 5, *n* = 19), and “work simulation, work training, and work conditioning” (Category 2, *n* = 17) (Table 3). When aggregated into broader domains, “RTW process coordination” was the most commonly reported domain (total *n* = 41), followed by “workplace interface and environmental adaptation” (total *n* = 39) and “work‐focused therapeutic intervention” (total *n* = 31). “Work capacity assessment” was also frequently described (*n* = 24), whereas “psychosocial support” appeared less frequently reported (*n* = 10) across the included studies (Table 3).

Across the PEO classification, the occupation and environment domains contained the largest number of OT role categories, particularly Category 1 (work participation–oriented assessment and job analysis), Category 5 (workplace evaluation and accommodations), and Category 6 (employer liaison, stakeholder meetings, and mediation). These categories were also most frequently reported in studies describing explicitly integrated therapeutic support. Category 7 (RTW planning, graded return, and follow‐up monitoring), although less frequently reported overall, was similarly more common in studies with explicitly integrated support than in the other therapeutic integration patterns (Table 3).

### 3.4. What Facilitators and Barriers to RTW Are Reported in the Literature? (RQ3)

Facilitators and barriers influencing RTW after stroke were identified across nine categories, which were further grouped into three broader domains: individual, workplace, and system/service factors (Table 4). To provide a comprehensive overview of factors influencing RTW after stroke, facilitators, barriers, and assessment approaches were analyzed across all included studies (*n* = 58), not only those reporting OT involvement.

**Table 4:** Facilitators and barriers to return to work after stroke.

Type	Category	Facilitators	*N*	Barriers	*N*
Individual factors	1. Personal factors	Functional capacity	7	Functional impairment (cognitive, physical, motor speed, ADL limitations)	28
		Fatigue management	7	Fatigue and reduced endurance	22
		Psychological adjustment and coping	7	Psychological challenges and identity disruption	12
		Self‐regulatory capacities	10	Limited insight and self‐regulatory capacity	6
		Work‐related competence and experience	13		
	2. Psychological factors	Family support and involvement	9	Insufficient psychosocial and mental health support	4
		Peer and community‐based support	7	Anxiety and fear related to health and work	3
		Ongoing emotional and guidance support	10	Social isolation and role strain	4
				Maladaptive family involvement	1
	3. Sociodemographic factors	Gender‐related characteristics	1	Financial strain and economic insecurity	10
		Socioeconomic resources	5	Pre‐existing socioeconomic vulnerability	7

Workplace factors	4. Workplace climate factors	Social support within the workplace	17	Negative workplace attitudes and unsupportive organizational culture	10
		Organizational culture and understanding	9	Limited workplace understanding of stroke and invisible impairments	15
		Workplace accommodation and assistive support	9	Structural employment pressures and productivity norms	7
		Workplace education and awareness	5		
	5. Job design factors	Workplace flexibility and graded return	18	High job demands and poor job–ability fit	9
		Role modification and task adjustment	9	Limited role options and employment flexibility	4
		Job matching and employment modification	8	Unstable or resource‐constrained work environments	6
		Lower physical job demands	6	Transportation and geographic access barriers	8
				Insufficient workplace feedback mechanisms	4

Sysyem/service factors	6. Rehabilitation support factors	Early and ongoing work‐focused support	19	Discontinuity and premature withdrawal of support	6
		Person‐centred and individualized goal‐directed process	10	Lack of structured rehabilitation pathways	6
		Work‐focused therapeutic interventions	17	Inadequate work‐focused assessment	3
				Poor individualization of support	2
	7. Capacity development factors	Professional education and RTW‐specific expertise	6	Insufficient professional education and RTW‐specific expertise	9
		Structured implementation frameworks	6	Implementation gaps in practice	5
		Workforce development and awareness promotion	5	Lack of structured RTW frameworks	4
	8. Coordination factors	Defined coordination roles and specialist involvement	14	Role ambiguity and unclear responsibilities	5
		Structured communication and information‐sharing systems	8	Service fragmentation and lack of coordination	12
		Intersectoral and multidisciplinary collaboration	14	Breakdowns in communication and information sharing	12
		Workplace‐engaged coordination and phased return mechanisms	16	Discontinuity and delayed support	9
	9. System and policy factors	Policy and insurance frameworks (insurance/benefits, legal framework, clear policies)	7	Regulatory and insurance constraints	8
		Service delivery models (private/home rehabilitation)	4	Administrative and structural system constraints	7
				System‐level resource and time limitations	11
				External system disruptions	2

At the individual factor level, the most frequently reported barriers were functional impairment (*n* = 28) and fatigue and reduced endurance (*n* = 22). Psychological challenges and identity disruption (*n* = 12) and financial strain and economic insecurity (*n* = 10) were also noted. Key facilitators within this domain included work‐related competence and prior experience (*n* = 13), self‐regulatory capacities (*n* = 10), and ongoing emotional support and guidance (*n* = 10), with family support and involvement reported slightly less frequently (*n* = 9).

Within the workplace domain, workplace flexibility and graded return schedules (*n* = 18) and social support within the workplace (*n* = 17) were the most frequently reported facilitators. Other facilitators included supportive organizational culture and understanding (*n* = 9), workplace accommodations or assistive resources (*n* = 9), and role modification and task adjustment (*n* = 9). Reported barriers in this domain included limited awareness of stroke and invisible impairments among employers (*n* = 15), negative workplace attitudes and unsupportive organizational culture (*n* = 10), and high job demands combined with poor job‐ability fit (*n* = 9).

At the system and service level, commonly reported facilitators included early and ongoing work‐focused support (*n* = 19), work‐oriented therapeutic interventions (*n* = 17), and a coordinated, workplace‐engaged phased return mechanism (*n* = 16). Reported barriers within this domain included fragmented services and lack of coordination across systems (*n* = 12), breakdowns in communication and information sharing (*n* = 12), and system‐level limitations such as constrained resources or insufficient time (*n* = 11).

### 3.5. Assessment Approaches Used in RTW Research

Assessment tools employed to evaluate stroke‐related difficulties affecting RTW are summarized in Table 5. Across the included studies (*n* = 58), assessments of work ability and work‐specific functional capacity were the most frequently reported (*n* = 29), followed closely by cognitive function assessments (*n* = 28) and physical function or activities of daily living assessments (*n* = 14). Other assessment types were reported less frequently. Notably, fatigue‐specific assessments were reported in only six studies. However, as shown in Table 5, fatigue was the second most frequently reported barrier to RTW (*n* = 22).

**Table 5:** Assessment approaches used in return‐to‐work research.

Assessment name	*N*
**Work and vocational outcomes**	**9**
RTW status/work outcomes	2
RTW process knowledge score (Max 8)	1
RTW questionnaire	1
RTW process indicators	2
Work readiness	3
**Work ability and work-specific functional assessment**	**29**
Assessment of work characteristics (AWC)	2
Work ability index (WAI)	1
Worker role interview (WRI)	1
General aptitude test battery (GATB)	1
Work sample test	2
Actual work performance assessment	6
Driving simulator	1
Functional capacity assessment	1
Job demands/job analysis	3
Work role assessment	1
Workplace assessment	6
Core work skill assessment	1
Evaluation of specific physical functions required for work	1
Repeated work ability evaluation	2
**Psychological/emotional assessment**	**10**
Generalized Anxiety Disorder‐7 (GAD‐7)	1
Hospital Anxiety and Depression Scale (HADS)	2
Patient Health Questionnaire‐9 (PHQ‐9)	1
General Self‐Efficacy Scale (GSES)	1
Depression Anxiety Stress Scales (DASS)	1
Assessment of the process of disability awareness	1
Work and Social Adjustment Scale	1
Tokyo University Egogram	1
Perceived competency score (Max 100%)	1
**Fatigue assessment**	**6**
Mental Fatigue Scale	3
Neurological Fatigue Index—Stroke, Work, and Social Adjustment	1
Fatigue self‐management assessment	2
**Cognitive function assessment**	**28**
Ranchos Amigos Level	2
Mini‐Mental State Examination (MMSE)	1
Montreal Cognitive Assessment (MoCA)	2
Oxford Cognitive Screen (OCS)	1
Revised Hasegawa′s Dementia Scale (HDS‐R)	2
Executive function assessment	3
Rivermead Behavioural Memory Test (RBMT)	2
Trail Making Test (TMT‐A/B)	2
Wechsler Adult Intelligence Scale–revised (WAIS‐R)	1
Kana Pick‐out Test	1
Hamamatsu Method High Level Cerebral Function Scale	1
AusTOM (language/speech/cognitive communication)	1
Checklist for cognitive and communication consequences of acquired brain injury	1
Classification of cognitive function based on neuropsychological assessment	1
Cognitive and affective self‐management	1
Subjective evaluation (e.g., executive function, memory, and social behavior)	1
Attention and memory assessment	2
Communication and interpersonal skills assessment	2
Academic skill assessment	1
**Physical function/ADL assessment**	**14**
Barthel Index (BI)	4
Brunnstrom recovery stage (BRS)	1
Functional Independence Measure (FIM)	2
Modified Rankin scale (mRS)	2
Modified Rivermead Mobility Index (MRMI)	1
National Institutes of Health Stroke Scale (NIHSS)	2
Nottingham Extended Activities of Daily Living Scale (NEADL)	1
Endurance assessment	1
**Stroke-specific assessment**	**5**
Stroke knowledge score (Max 7)	1
Stroke‐related impairment assessment	1
Stroke Impact Scale (SIS)	2
Post‐stroke checklist	1
**Quality of life and participation**	**7**
Canadian Occupational Performance Measure (COPM)	2
Community integration questionnaire (CIQ)	1
EuroQol‐5D (EQ‐5D)	2
Stroke‐specific QoL	1
Participation assessment scale	1

*Note:* Bold text indicates the category name that summarizes the individual assessment measures listed below in regular font. Bold numbers in the right column indicate the total number of assessment measures included in that category.

## 4. Discussion

### 4.1. Overview of the Principal Findings

This ScR explored how therapeutic support is incorporated into RTW processes after stroke and examined the OT‐related roles reported within these processes.

First, the reviewed studies demonstrated varying levels of therapeutic integration within RTW processes. While some studies described therapeutic interventions that were completed before work resumption, others reported ongoing therapeutic support delivered alongside RTW or explicitly integrated with workplace demands. These findings suggest that therapeutic support is incorporated into RTW processes in diverse ways and may reflect differences in how WTB is addressed in practice.

Second, a wide range of OT‐related roles was identified across the included studies. Ten categories of OT roles were extracted and organized into five broad domains representing key areas of OT involvement in RTW support: “work capacity assessment,” “work‐focused therapeutic intervention,” “workplace interface and environmental adaptation,” “RTW process coordination,” and “psychosocial support.” Together, these roles reflect OT contributions at the individual, workplace, and system levels of RTW support.

Third, facilitators and barriers to RTW were identified across individual, workplace, and system/service domains. Frequently reported barriers included functional impairment, fatigue and reduced endurance, and limited workplace understanding of stroke‐related difficulties. In contrast, frequently reported facilitators included workplace flexibility, social support, and coordinated work‐focused rehabilitation.

Finally, a mismatch was observed between the barriers reported in the literature and the assessment approaches used in RTW research. Although fatigue and reduced endurance were frequently identified as barriers to RTW, fatigue‐specific assessments were reported less frequently than other assessment categories.

### 4.2. Structural and Therapeutic Integration Patterns in RTW Support (RQ1)

The findings suggest that therapeutic support is incorporated into RTW processes after stroke in different ways. These differences may reflect how rehabilitation and employment support are organized and coordinated within each setting. Previous studies have suggested that limited coordination between healthcare and workplace services can hinder RTW, whereas closer collaboration may support workplace accommodations and sustained work participation [1, 4, 19, 20].

Previous research has shown that returning to work after stroke does not necessarily mark the end of work‐related challenges. Stroke survivors may continue to experience difficulties related to fatigue, cognitive demands, workplace expectations, and long‐term work sustainability, even after successful work resumption [21]. From a WTB perspective, ongoing therapeutic support may provide opportunities to address challenges that emerge after work resumption [22]; approaches that support the coexistence of treatment and work may become increasingly important.

Another notable finding was the predominance of qualitative research. Most studies focused on describing experiences, support processes, and perceived barriers, whereas quantitative and intervention studies were less common. This pattern suggests that research on RTW support after stroke remains at a relatively early stage, and further quantitative and intervention‐focused research is needed to evaluate the effectiveness of different support approaches.

### 4.3. OT Roles Within RTW Support Processes (RQ2)

The findings of this review suggest that OT involvement in RTW support extends beyond individual rehabilitation and often includes workplace‐focused assessment as well as coordination across service systems. Across the included studies, OT roles were described in 10 categories, which could be broadly grouped into domains related to work capacity assessment, work‐focused intervention, workplace interface, and RTW process coordination. Among these, “work participation–oriented assessment and job analysis,” “employer liaison and stakeholder mediation,” and “workplace evaluation and accommodations” were particularly prominent. These roles suggest that OT involvement in RTW support frequently involves linking workers′ functional capacities with job demands while facilitating interaction between healthcare providers, employers, and other stakeholders, which may be relevant to supporting WTB in practice. Previous research has shown that employers often require information, communication, and clinical support when accommodating workers after stroke [23]. This may help explain the prominence of workplace‐focused and coordination‐related OT roles in the reviewed studies.

These findings are consistent with the PEO perspective, which emphasizes the interaction among workers′ abilities, work activities, and workplace environments. Workplace accommodations and employer engagement can help improve the fit between workers′ abilities and job requirements following stroke and have been identified as important components of effective RTW interventions [4, 24]. This may help explain why workplace‐focused and coordination‐related OT roles were frequently reported in the reviewed studies. In Japan, WTB coordinators facilitate communication between healthcare providers and employers. Several OT roles identified in this review, particularly employer liaison and RTW coordination, reflected similar activities.

### 4.4. Stroke‐Specific Determinants and Gaps in Assessment (RQ3)

Poststroke fatigue was frequently identified as a barrier to sustained work participation in the included studies (Table 5). Previous research has shown that fatigue often persists after stroke and can affect both work performance and long‐term work participation [25, 26]. Cognitive and emotional challenges have also been reported to influence work performance and adaptation to workplace demands [27–29].

When these determinants were compared with the assessment approaches used in the reviewed studies, a contrasting pattern emerged. Functional impairments—the most frequently reported barriers to RTW (*n* = 28)—were commonly evaluated using cognitive and physical function assessments (*n* = 28). In contrast, fatigue—despite being the second most frequently reported barrier (*n* = 22)—was assessed in relatively few studies (*n* = 6). Many studies focused on evaluating work ability, cognitive function, or participation outcomes, whereas standardized fatigue assessments were rarely reported. This suggests that fatigue remains underrepresented in RTW‐focused evaluations despite being one of the most commonly reported barriers to work participation. Addressing this gap may improve the identification of work‐related difficulties after RTW and support more targeted rehabilitation strategies.

### 4.5. Implications for OT Practice and System Development

The findings of this review suggest that OT involvement in RTW support extends beyond individual rehabilitation. OTs were often involved in assessing work demands, supporting workplace adjustments, and coordinating communication between healthcare providers and employers. These activities may support the integration of work and treatment during the RTW process. The review also identified a discrepancy between reported barriers and assessment practices. Poststroke fatigue was frequently reported as a barrier to work participation, but fatigue‐specific assessments were used in relatively few studies. This suggests that an important challenge affecting work participation may not be routinely assessed in current RTW support.

Although coordination‐related functions were identified across the included studies, explicit descriptions of formally defined coordination roles were limited. This may reflect variations in terminology, role definitions, and the organization of rehabilitation and employment support services across countries. Such differences may influence how coordination functions are implemented and how work and treatment are integrated within RTW processes. Therefore, the applicability of the present findings may vary across service contexts, and the specific nature of coordination practices may not be fully captured through document‐based analysis alone. Further qualitative research is needed to better understand how these coordination functions are enacted in practice.

### 4.6. Methodological Considerations and Limitations

This review has several limitations. First, as an ScR, it did not include a formal appraisal of methodological quality, and the included studies represented a range of heterogeneous designs. Therefore, the findings should be interpreted as descriptive mappings of structural patterns rather than evaluations of intervention effectiveness. Second, variations in terminology, service organization, and policy frameworks across countries may have influenced the interpretation of therapeutic integration and coordination functions within RTW support. Because rehabilitation and employment support services are organized differently across countries, the applicability of the findings may vary across service contexts. Third, the classification of therapeutic integration levels and OT role domains relied on explicit textual descriptions. Although coding decisions were made through reviewer consensus, variability in reporting detail may have influenced categorization. Finally, OT involvement was identified only when explicitly reported in the included studies. As a result, OT contributions within multidisciplinary RTW programs may be underrepresented. The OT roles identified in this review should therefore be interpreted as explicitly reported activities rather than a complete representation of OT practice within RTW support.

## 5. Conclusions

This ScR showed that therapeutic support was incorporated into RTW processes in different ways across the reviewed studies. OT involvement extended beyond individual rehabilitation and included workplace assessment, workplace adjustment, and coordination with employers and other stakeholders. Poststroke fatigue was frequently reported as a barrier to work participation, but fatigue‐specific assessments were rarely used. Coordination‐related activities were commonly described, although formal coordination roles were seldom reported. Differences in service systems across countries may also influence how work and treatment are integrated during the RTW process. These findings contribute to current understanding of RTW support after stroke and may inform future research and service development.

## Author Contributions

R.M. contributed to the conceptualization, methodology, literature search, screening, data extraction, qualitative synthesis, and manuscript drafting and served as the corresponding author. Both R.M. and P.B. independently screened records and extracted data. P.B. provided methodological oversight, supported data extraction, guided interpretation, validated the synthesis, and contributed to manuscript drafting.

## Funding

This research was supported by a grant from the Tokyo Metropolitan University′s competitive inclined research funds for the 2023 fiscal year.

## Disclosure

All authors reviewed and approved the final version of the manuscript.

## Conflicts of Interest

The authors declare no conflicts of interest.

## Supporting information


**Supporting Information** Additional supporting information can be found online in the Supporting Information section. Table S1. Data extraction sheet used in this review.

## Data Availability

The data supporting the findings of this study are available from the corresponding author upon reasonable request.
